# Big Food, Food Systems, and Global Health

**DOI:** 10.1371/journal.pmed.1001242

**Published:** 2012-06-19

**Authors:** David Stuckler, Marion Nestle

**Affiliations:** 1 Department of Sociology, University of Cambridge, Cambridge, United Kingdom; 2 Department of Public Health & Policy, London School of Hygiene & Tropical Medicine, London, United Kingdom; 3 Department of Nutrition, Food Studies, and Public Health, New York University, New York, New York, United States of America; 4 Department of Nutritional Sciences, Cornell University, Ithaca, New York, United States of America

## Abstract

In an article that forms part of the PLoS Medicine series on Big Food, guest editors David Stuckler and Marion Nestle lay out why more examination of the food industry is necessary, and offer three competing views on how public health professionals might engage with Big Food.

*This article was commissioned for the* PLoS Medicine *series on Big Food that examines the activities and influence of the food and beverage industry in the health arena.*

As the *PLoS Medicine* series on Big Food (www.ploscollections.org/bigfood) kicks off, let's begin this Essay with a blunt conclusion: Global food systems are not meeting the world's dietary needs [1]. About one billion people are hungry, while two billion people are overweight [2]. India, for example, is experiencing rises in both: since 1995 an additional 65 million people are malnourished, and one in five adults is now overweight [3],[4]. This coexistence of food insecurity and obesity may seem like a paradox [5], but over- and undernutrition reflect two facets of malnutrition [6]. Underlying both is a common factor: food systems are not driven to deliver optimal human diets but to maximize profits. For people living in poverty, this means either exclusion from development (and consequent food insecurity) or eating low-cost, highly processed foods lacking in nutrition and rich in sugar, salt, and saturated fats (and consequent overweight and obesity).

To understand who is responsible for these nutritional failures, it is first necessary to ask: *Who rules global food systems?* By and large it's “Big Food,” by which we refer to multinational food and beverage companies with huge and concentrated market power [7],[8]. In the United States, the ten largest food companies control over half of all food sales [9] and worldwide this proportion is about 15% and rising. More than half of global soft drinks are produced by large multinational companies, mainly Coca-Cola and PepsiCo [10]. Three-fourths of world food sales involve processed foods, for which the largest manufacturers hold over a third of the global market [11]. The world's food system is not a competitive marketplace of small producers but an oligopoly. What people eat is increasingly driven by a few multinational food companies [12].

Virtually all growth in Big Food's sales occurs in developing countries [13] (see Figure 1). The saturation of markets in developed countries [14], along with the lure of the 20% of income people spend on average on food globally, has stimulated Big Food to seek global expansion. Its rapid entry into markets in low- and middle-income countries (LMICs) is a result of mass-marketing campaigns and foreign investment, principally through takeovers of domestic food companies [15]. Trade plays a minimal role and accounts for only about 6% of global processed food sales [15]. Global producers are the main reason why the “nutrition transition” from traditional, simple diets to highly processed foods is accelerating [16],[17].

**Figure 1:**
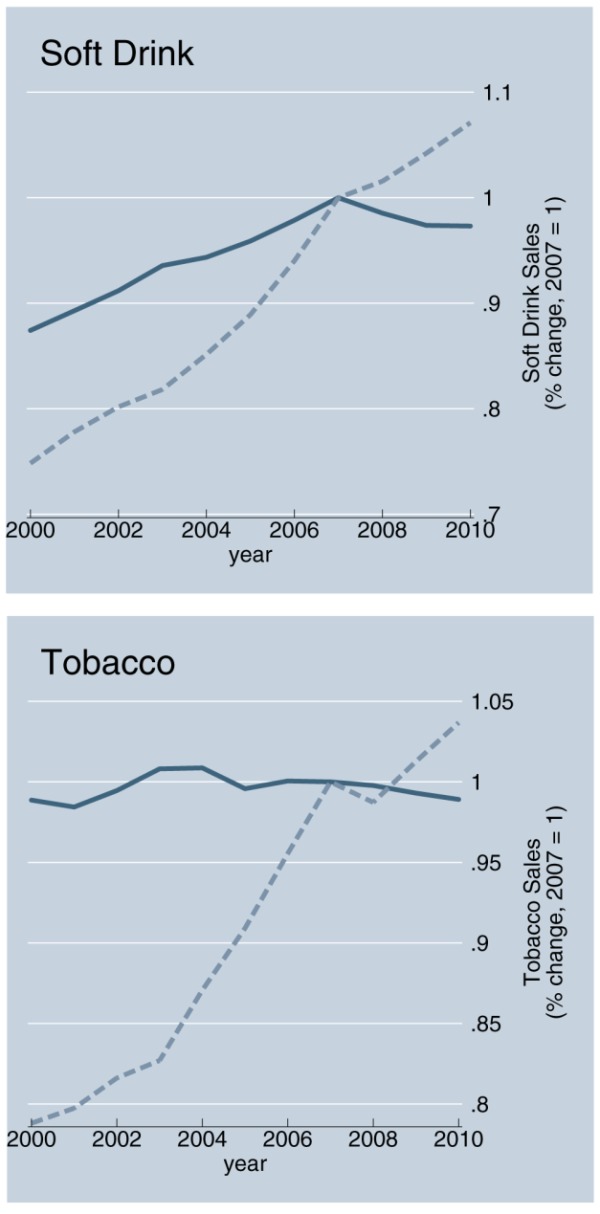
Growth of Big Food and Big Tobacco sales in developing countries: An example. Shaded blue line is developed countries, dashed grey line is developing countries. Source: Passport Global Market Information Database: EuroMonitor International, 2011 [12].

Big Food is a driving force behind the global rise in consumption of sugar-sweetened beverages (SSBs) and processed foods enriched in salt, sugar, and fat [13]. Increasing consumption of Big Food's products tracks closely with rising levels of obesity and diabetes [18]. Evidence shows that SSBs are major contributors to childhood obesity [19],[20], as well as to long-term weight-gain, type 2 diabetes, and cardiovascular disease [21],[22]. Studies also link frequent consumption of highly processed foods with weight gain and associated diseases [23].

Of course, Big Food may also bring benefits—improved economic performance through increased technology and know-how and reduced risks of undernutrition—to local partners [24]. The extent of these benefits is debatable, however, in view of negative effects on farmers and on domestic producers and food prices [25].

## Public Health Response to Big Food: A Failure to Act

Public health professionals have been slow to respond to such nutritional threats in developed countries and even slower still in developing countries. Thanks to insights from tobacco company documents, we have learned a great deal about how this industry sought to avoid or flout public health interventions that might threaten their profits. We now have considerable evidence that food and beverage companies use similar tactics to undermine public health responses such as taxation and regulation [26],[27],[28],[29], an unsurprising observation given the flows of people, funds, and activities between Big Tobacco and Big Food. Yet the public health response to Big Food has been minimal.

We can think of multiple reasons for the failure to act [30]. One is the belated recognition of the importance of obesity to the burden of disease in LMICs [13]. The 2011 Political Declaration of the United Nations High-Level Meeting on Prevention and Control of Non-communicable Diseases (NCDs) recognized the urgent case for addressing the major avoidable causes of death and disability [31], but did not even mention the roles of agribusiness and processed foods in obesity. Despite evidence to the contrary, some development agencies continue to view obesity as a “disease of affluence” and a sign of progress in combating undernutrition [32].

A more uncomfortable reason is that action requires tackling vested interests, especially the powerful Big Food companies with strong ties to and influence over national governments. This is difficult terrain for many public health scientists. It took five decades after the initial studies linking tobacco and cancer for effective public health policies to be put in place, with enormous cost to human health. Must we wait five decades to respond to the similar effects of Big Food?

If we are going to get serious about such nutritional issues, we must make choices about how to engage with Big Food. Whether, and under what circumstances, we should view food companies as “partners” or as part of the solution to rising rates of obesity and associated chronic diseases is a matter of much current debate, as indicated by the diverse views of officials of PepsiCo and nutrition scientists [24],[27],[28],[33],[34].

## Engaging with Big Food—Three Views

We see three possible ways to view this debate. The first favors voluntary self-regulation, and requires no further engagement by the public health community. Those who share this view argue that market forces will self-correct the negative externalities resulting from higher intake of risky commodities. Informed individuals, they say, will choose whether to eat unhealthy foods and need not be subjected to public health paternalism. On this basis, UN secretary-general Ban Ki Moon urged industry to be more responsible: “I especially call on corporations that profit from selling processed foods to children to act with the utmost integrity. I refer not only to food manufacturers, but also the media, marketing and advertising companies that play central roles in these enterprises” [35]. Similarly, the UK Health Minister recently said: “the food and drinks industry should be seen, not just as part of the problem, but part of the solution…An emphasis on prevention, physical activity and personal and corporate responsibility could, alongside unified Government action, make a big difference” [36].

The second view favors partnerships with industry. Public health advocates who hold this view may take jobs with industry in order to make positive changes from within, or actively seek partnerships and alliances with food companies. Food, they say, is not tobacco. Whereas tobacco is demonstrably harmful in all forms and levels of consumption, food is not. We can live without tobacco, but we all must eat. Therefore, this view holds that we must work with Big Food to make healthier products and market them more responsibly.

The third approach is critical of both. It recognizes the inherent conflicts of interest between corporations that profit from unhealthy food and public health collaborations. Because growth in profit is the primary goal of corporations, self-regulation and working from within are doomed to fail. Most proponents of this viewpoint support public regulation as the only meaningful approach, although some propose having public health expert committees set standards and monitor industry performance in improving the nutritional quality of food products and in marketing the products to children.

We support the critical view, for several reasons. First, we find no evidence for an alignment of public health interest in curbing obesity with that of the food and beverage industry. Any partnership *must* create profit for the industry, which has a legal mandate to maximize wealth for shareholders. We also see no obvious, established, or legitimate mechanism through which public health professionals might increase Big Food's profits.

Big Food attains profit by expanding markets to reach more people, increasing people's sense of hunger so that they buy more food, and increasing profit margins through encouraging consumption of products with higher price/cost surpluses [28]–[31],[37]. Industry achieves these goals through food processing and marketing, and we are aware of no evidence for health gains through partnerships in either domain. Although in theory minimal processing of foods can improve nutritional content, in practice most processing is done so to increase palatability, shelf-life, and transportability, processes that reduce nutritional quality. Processed foods are not necessary for survival, and few individuals are sufficiently well-informed or even capable of overcoming marketing and cost hurdles [38]. Big Food companies have the resources to recruit leading nutritional scientists and experts to guide product development and reformulation, leaving the role of public health advisors uncertain.

To promote health, industry would need to make and market healthier foods so as to shift consumption away from highly processed, unhealthy foods. Yet, such healthier foods are inherently less profitable. The only ways the industry could preserve profit is either to undermine public health attempts to tax and regulate or to get people to eat more healthy food while continuing to eat profitable unhealthy foods [33],[39]. Neither is desirable from a nutritional standpoint. Whereas industry support for research might be seen as one place to align interests, studies funded by industry are 4- to 8-fold more likely to support conclusions favorable to the industry [40].

Our second reason to support the critical view has to do with the “precautionary principle” [41]. Because it is unclear whether inherent conflicts of interest can be reconciled, we favor proceeding on the basis of evidence. As George Orwell put it, “saints should always be judged guilty until they are proved innocent.” We believe the onus of proof is on the food industry. If food companies can rigorously and independently establish self-regulation or private–public partnerships as improving both health and profit, these methods should be extended and replicated. But to date self-regulation has largely failed to meet stated objectives [42],[43],[44],[45],[46],[47], and instead has resulted in significant pressure for public regulation. Kraft's decision to ban trans fats, for example, occurred under pressure of lawsuits [48]. If industry believed that self-regulation would increase profit, it would already be regulating itself.

We believe the critical view has much to offer. It is a model of dynamic and dialectic engagement. It will increase pressures on industry to improve health performance, and it will encourage those who are sympathetic to the first or second views to effect change from within large food and beverage companies.

Public health professionals must recognize that Big Food's influence on global food systems is a problem, and do what is needed to reach a consensus about how to engage critically. The Conflicts of Interest Coalition, which emerged from concerns about Big Food's influence on the U.N. High-Level Meeting on NCDs, is a good place to start [29],[49]. Public health professionals must place as high a priority on nutrition as they do on HIV, infectious diseases, and other disease threats. They should support initiatives such as restrictions on marketing to children, better nutrition standards for school meals, and taxes on SSBs. The central aim of public health must be to bring into alignment Big Food's profit motives with public health goals. Without taking direct and concerted action to expose and regulate the vested interests of Big Food, epidemics of poverty, hunger, and obesity are likely to become more acute.

## Abbreviations

LMIC: low- and middle-income country
SSB: sugar-sweetened beverage
